# Treatment of Supracondylar Femur Fractures in Cats with EFECE System

**DOI:** 10.1002/vms3.70691

**Published:** 2025-11-18

**Authors:** Ali Gülaydin, Nihat Şindak, Mustafa Barış Akgül, Onur Yildirim, Emre Karadeniz, Müzzemil Hattap Soysal

**Affiliations:** ^1^ Department of Surgery Faculty of Veterinary Medicine Siirt University Siirt Türkiye; ^2^ Department of Orthopaedics and Traumatology Faculty of Medicine Kocaeli University İzmit Kocaeli Türkiye; ^3^ Department of Veterinary Surgery Institute of Health Sciences Siirt University Siirt Türkiye

**Keywords:** cat, EFECE, femur, fractures, supracondylar

## Abstract

**Background:**

Distal supracondylar femur fractures are common orthopaedic injuries in cats, requiring stable fixation for optimal healing. Traditional fixation methods have limitations, leading to the exploration of alternative techniques. The EFECE system, a novel fracture fixation method, aims to provide effective stabilization while minimizing complications.

**Objectives:**

This study evaluates the clinical and radiological outcomes of distal supracondylar femur fracture treatment in cats using the EFECE system.

**Methods:**

Eight cats diagnosed with distal supracondylar femur fractures were included. All cases were closed fractures treated using a limited open reduction technique followed by fixation with the EFECE system. Fracture healing time and complication rates were assessed through clinical and radiographic evaluations.

**Results:**

All fractures achieved complete healing, with no major complications observed. The mean healing time was 42.8 days (range: 35–56 days). Minor complications were observed in three out of the eight cats included in the study. Weight‐bearing, behaviour and radiographic findings confirmed effective stabilization and compression of the fracture line using the EFECE system.

**Conclusions:**

The EFECE system provides a reliable and minimally invasive option for treating distal supracondylar femur fractures in cats, ensuring stable fixation and favourable clinical outcomes. Further studies with larger sample sizes are recommended to validate these findings.

## Graphical abstract

The EFECE system demonstrated effective and stable fixation in feline distal supracondylar femur fractures, with complete healing observed in all cases and minimal complications. This novel method offers a promising, minimally invasive alternative for fracture management in cats.

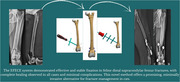

## 1 Introduction

Supracondylar fracture fixation is surgically challenging due to the short distal fragment and limited bone stock (Harasen 2002; Longley et al. 2018). In the treatment of these fractures, methods such as intramedullary cross pinning (Altunatmaz et al. 2017; figure‐of‐eight stainless steel wire (Spångberg et al. 2019), arrow pin (Rathnadiwakara et al. 2020) and internal fixation with crossed K‐wires (Rubinos and Meeson 2022) can be used, while a more rigid fixation with a plate (Field et al. 2018), external fixator (Gülaydın and Alkan 2024) or angled stable interlocking nail is recommended (Marturello et al. 2021). In intramedullary pinning procedures, failure to provide rigid fixation of the fragments due to lack of compression between the fracture fragments after reduction and migration of the intramedullary pin used are considered serious complications (Bondonny et al. 2024; Karadeniz 2021). Due to the caudal slope of the femoral condyle, it may be recommended to use anatomical condylar J‐shaped plates that allow the insertion of at least three screws into the distal fragment in the treatment of related fractures with plates (Field et al. 2018). As an alternative, veterinary reconstruction plates, pearl‐locking plates or other similar systems can be used. However, these plate systems are known to pose significant complications such as lack of sufficient strength or stiffness to act as a bridge, high implant failure, risk of skin closure prevention, loss of range of motion and new bone fractures (DeCamp et al. 2016; Field et al. 2018; Macias et al. 2006). In supracondylar fractures, hybrid external fixators (Gülaydın and Alkan 2024) and distal miniature circular fixators (IMEX) are used for rigid fixation (Farese et al. 2002). The major disadvantages of these systems are pin‐site infections that may occur due to the moving skin and soft tissue around the femur (Beever et al. 2017).

**FIGURE 1:**
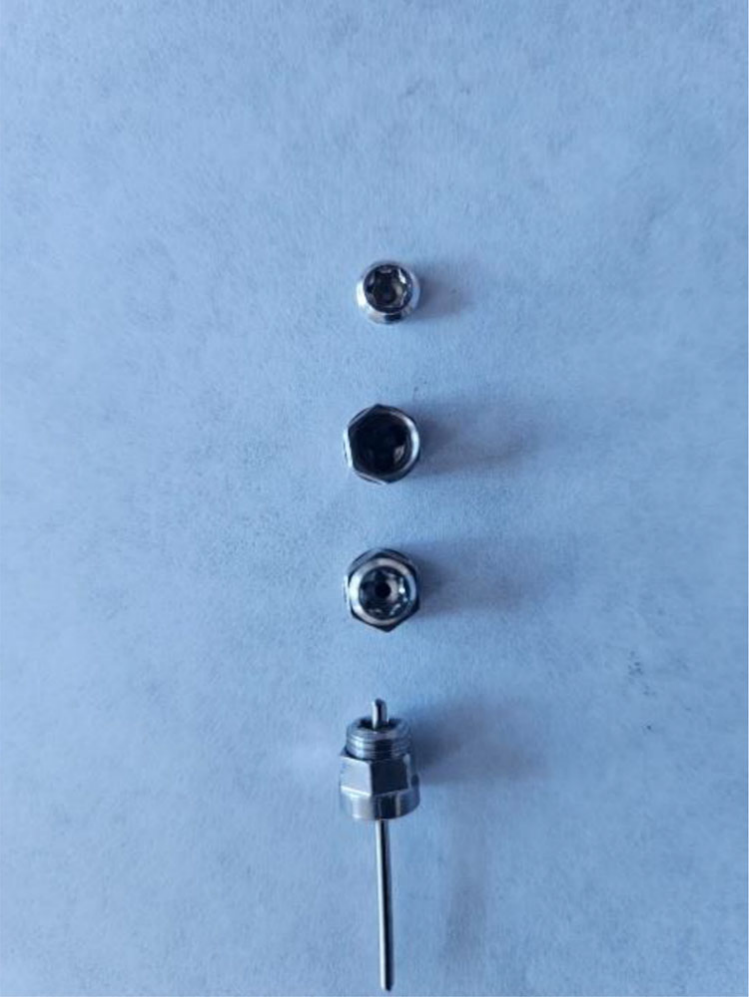
The locking mechanism of the EFECE wire holder.

Considering the treatment options for supracondylar femur fractures and the possible problems, the pursuit of a new treatment system has begun. Therefore, it was decided to apply the EFECE system designed by Karadeniz (2021). The system is a new fracture fixation method consisting of an EFECE wire holder, EFECE wire and EFECE hand tools. The EFECE wire holder is composed of two cylinder‐shaped parts, each with a central hole for the EFECE wire and interconnected by grooves. The upper part functions as a lid. The lower part is a system with a locking mechanism since three balls are inserted into three funnel‐shaped grooves (Figure 1). When moving forward, this mechanism allows the balls to travel towards the wider base of the funnel‐shaped groove; however, when moving backwards, the balls become stuck in the narrower part of the funnel‐shaped groove, preventing the EFECE wire from moving. This feature is crucial for maintaining fracture fixation. Furthermore, since the EFECE system provides cortical retention with retainers in the bone, there is no issue with the removal of the retainers. Removal of the retainers after fracture treatment results in less bone and surrounding soft tissue damage, and this process can be easily accomplished with magnets. Furthermore, it is emphasised that the system will bring a brand‐new perspective to the existing surgical solutions since it is applied percutaneously and provides compression through thin wires (Karadeniz 2021).

Previous studies have reported the use of this implant system for tibial plateau fracture simulation, as well as in human cadaveric patellar fracture treatments (Karadeniz and Keskinoz 2020). However, based on our comprehensive literature review, there appear to be no published studies documenting the in vivo application of these implants in live subjects. Therefore, this study represents the first reported use of this implant system in veterinary medicine for in vivo fracture repair.

## 2 Methods

### 2.1 Inclusion Criteria

This study included eight cats of various breeds, ages and sexes brought to the Animal Health Practice and Research Hospital at XXX University between January 2022 and July 2024. These cats presented with complaints of severe lameness and were diagnosed with distal femur fractures based on clinical and radiographic evaluations. Only cases with no systemic diseases identified were included. Written consent was obtained from the owners of the patients before initiating treatment, in accordance with hospital regulations.

Ethical approval for this study was obtained from the Siirt Üniversitesi Animal Researches Local Ethics Committee 26/11/2021, under veterinary investigation number 2021/04/28. All owners were informed about the novel nature of the implants and potential risks, and written informed consent was obtained prior to inclusion of the animals in the study.

### 2.2 Pre‐Operative Management

All patients underwent a comprehensive preoperative general examination, including haematological evaluations using the Mindray BC‐60R Vet (Hasvet, Antalya, Türkiye). Prophylactic parenteral antibiotics (20 mg/kg ceftriaxone; Unacefin 0.5 g, Yavuz İlaç, Istanbul, Türkiye) were administered until surgery. The patients were hospitalised until the surgical procedure, which was performed within 1–2 days post‐admission. Radiographic confirmation of the fracture and treatment planning were performed using two‐view (anteroposterior and mediolateral) x‐rays of the affected extremity.

### 2.3 Retrieved Data

The retrieved data included fracture aetiology, fracture classification, time from trauma to surgical procedure, osteosynthesis material, physical examination findings (including neurological status), surgical technique, post‐operative complications and recovery time.

Fractures were classified according to the level of comminution using a modified version of the Winquist and Hansen system (Winquist and Hansen 1980; Gall et al. 2022). Complications were classified into minor (requiring no medical or surgical treatment), major (requiring medical or surgical intervention) and catastrophic (resulting in permanent and unacceptable limb dysfunction leading to amputation) categories (Cook et al. 2010). All complication assessments and outcome evaluations were performed by the same investigator to ensure consistency.

### 2.4 Surgical Technique

After induction of anaesthesia with 4 mg/kg propofol (Dormofol, Vem, Kapaklı, Tekirdağ, Türkiye), the surgical site was shaved and disinfected prior to surgery. Anaesthesia was maintained with a closed‐circuit anaesthesia device (SMS 2000 Classic Vent‐V, SMS Tıbbi Cihaz, Elek. Elekt. İnş. Öğr. Turz. Oto San. ve Tic. Ltd. Şti. Ltd. Şti. Ankara, Türkiye) with 2% sevoflurane (Sevorane, Abbott, Italy). The patients were placed on the operating table in the lateral position, keeping the affected extremity facing upwards. In all cases, open reduction was preferred due to the presence of severe dislocation at the fracture line and the necessity of achieving precise anatomical alignment, particularly in fractures near the joint. The fracture line was routinely reached using a limited incision (2–3 cm) open reduction technique (lateral parapatellar approach and arthrotomy were performed; when necessary, this approach was extended proximally towards the distal shaft of the femur) (Bondonny et al. 2024). The fragments were reduced. The patella was restored to its anatomical position, and the EFECE system was applied.

The EFECE system was applied according to the following steps.

**FIGURE 2:**
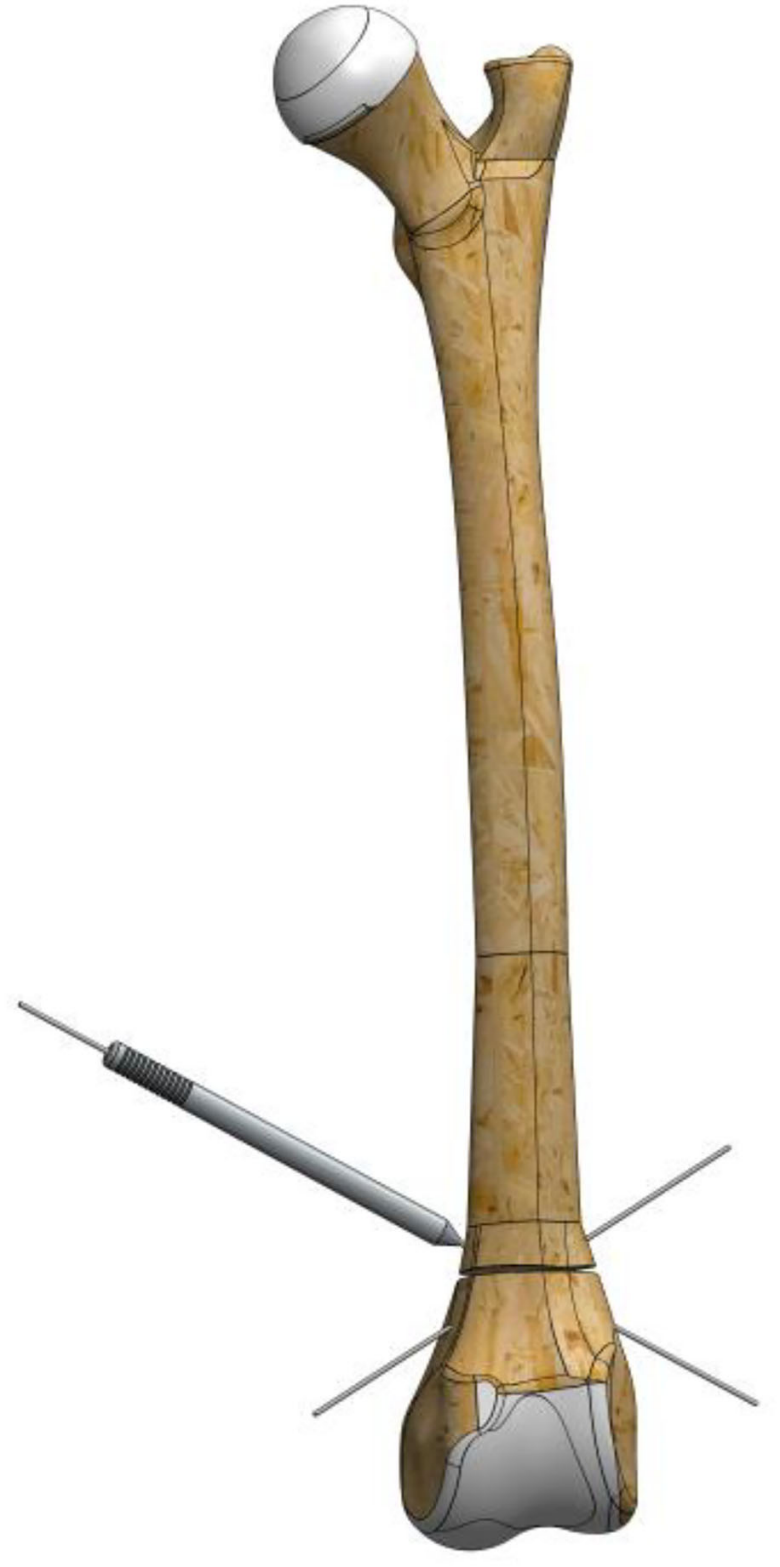
A tenon (sleeve) inserted through the wire and secured to the bone for stabilization.

**FIGURE 3:**
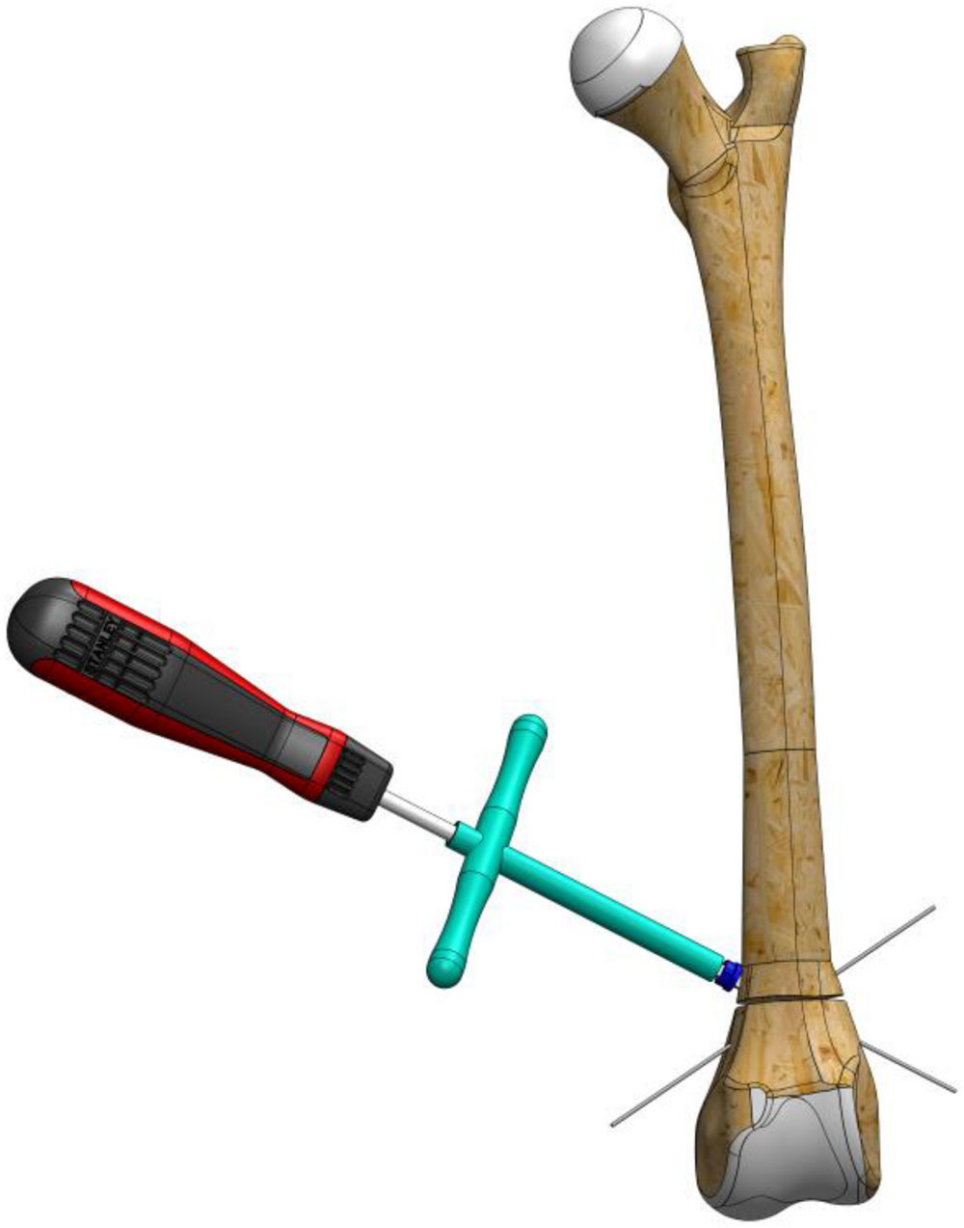
The screwdriver loads the EFECE wire holder while the external grasper is driven through the Kirschner wire to the bone. The screwdriver is tightened while keeping the EFECE external grasper stationary.

**FIGURE 4:**
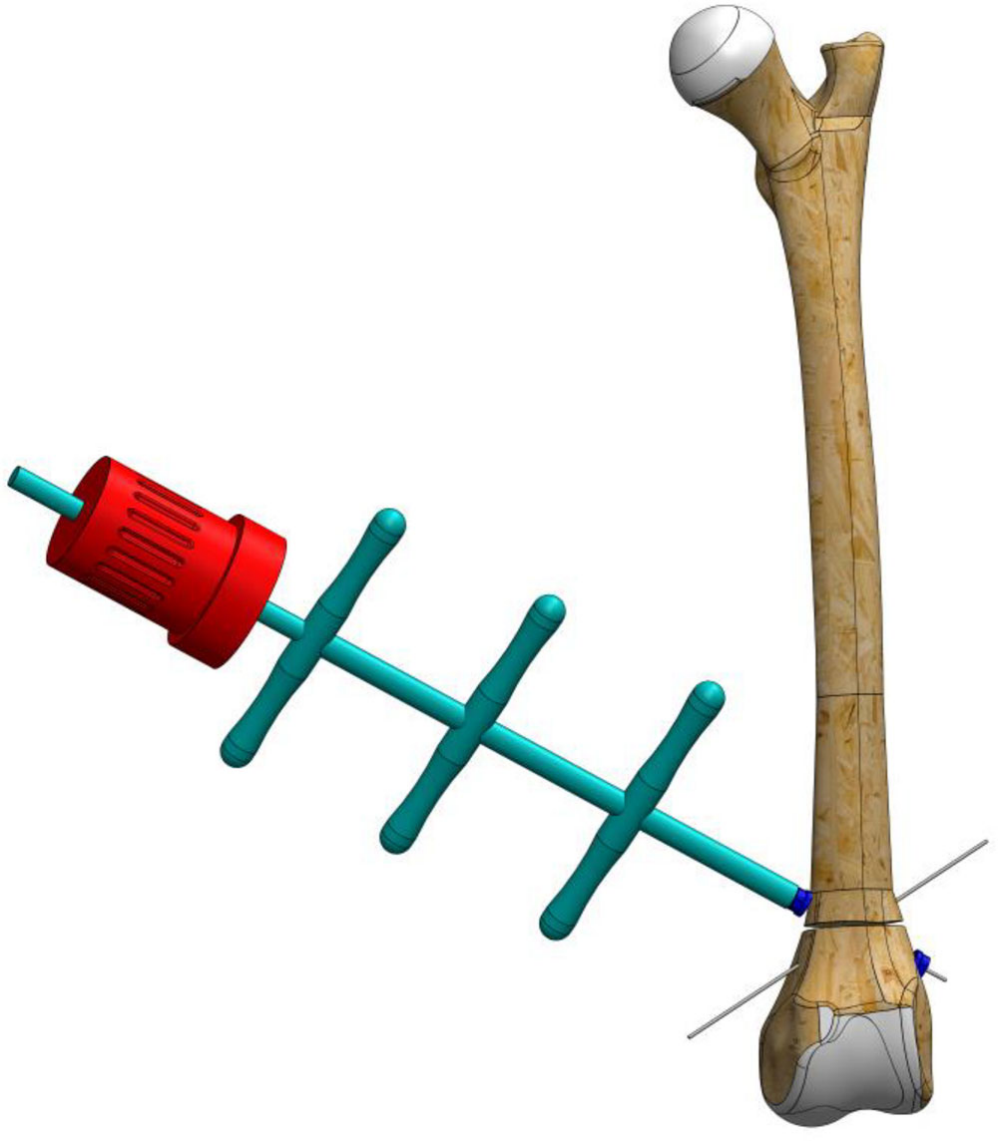
The EFECE wire tensioning device was mounted on the screwdriver over the wire, capturing and tensioning the EFECE wire to achieve the desired level of compression at the fracture line.

**FIGURE 5:**
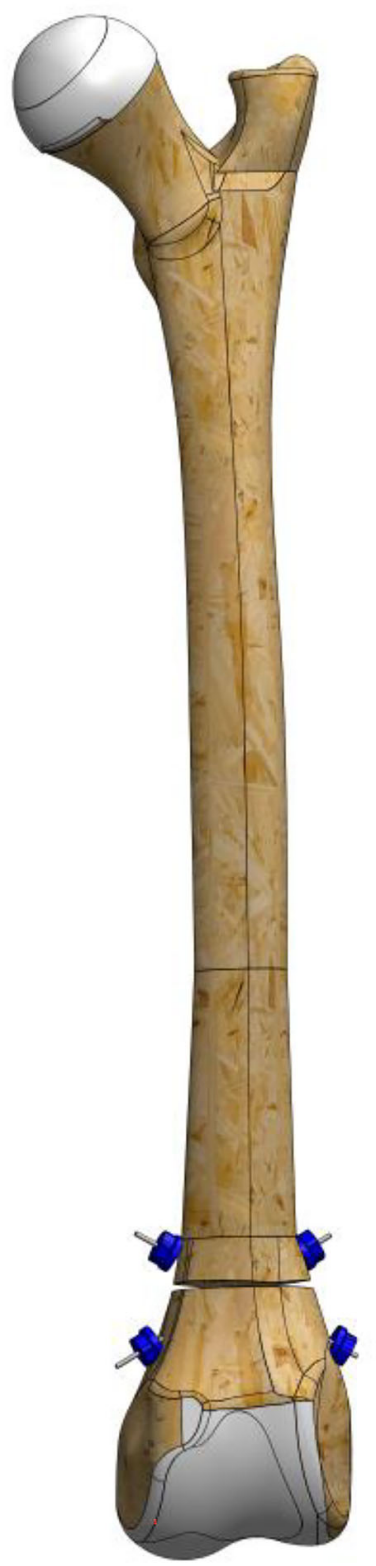
The excess part of the wires was cut off using the EFECE wire‐cutting tool.

- To stabilize the fracture line, a 1.2 mm Kirschner wire (manufactured with appropriate stiffness for the ball bearings of the EFECE wire holder) was first placed from the cranio‐distal aspect of the supracondylaris lateralis to the caudo‐proximal aspect of the corpus, followed by another placement from the cranio‐distal aspect of the supracondylaris medialis to the caudo‐proximal aspect of the corpus in a transcortical manner.
- A tenon (sleeve) was driven through the wire to the bone (Figure 2).
- The working cannula was driven through the tenon to the bone, and the tenon was removed.
- The screwdriver loaded the EFECE wire holder, and the external grasper was driven through the Kirschner wire to the bone. The screwdriver was tightened while the EFECE external grasper was held still (Figure 3).
- The working cannula was removed by pulling the external catcher and screwdriver.
- Operations ‘b’, ‘c’ and d were repeated at the other end of the EFECE wire, but the EFECE wire holder was not tightened.
- The EFECE wire tensioning device was mounted on the screwdriver over the wire. The EFECE wire was captured and tensioned to achieve the desired level of compression at the fracture line (Figure 4).
- While holding the EFECE external grasper stationary, the screwdriver was tightened and the EFECE wire tensioning device was loosened and removed.
- The working cannula was removed by pulling the external grabber and screwdriver.
- Excess part of wires was cut off with the EFECE wire‐cutting tool (Figure 5).
- The above procedures were repeated for the second EFECE wire (Karadeniz 2021).
- The incision site was closed, and the surgery was completed.

### 2.5 Post‐Operative Control and Care

The functions of the vascular and muscular structures of the affected extremities were checked by detailed clinical examination on the first post‐operative day. All patients were scheduled to be administered 20 mg/kg ceftriaxone (Unacefin 0.5 g, Yavuz İlaç, Istanbul, Türkiye) intramuscularly twice daily for 10 days post‐operatively. The patients were treated with 0.3 mg/kg meloxicam (Meloxicam, Bavet, Tuzla, Istanbul, Türkiye) for 3 days to relieve pain and inflammation.

### 2.6 Clinical Outcome Assessment

During the post‐operative period, criteria such as the use of the affected extremity, the presence of pain and oedema, joint functions and the presence of regional muscle and tendon contractures were reviewed. The findings of the cases were graded according to the functional and aesthetic diagnosis described by Rovesti (Table 1) (Rovesti et al. 2007).

**TABLE 1:** Functional and aesthetic grading of cases in the post‐operative period.

Grade	Lameness condition	Extremity appearance
Excellent	Normal gait, no lameness or pain.	Normal appearance
Good	Normal gait, mild lameness in the extremities	Normal appearance
Moderate	Mild or moderate lameness	Imperfect appearance
Thin	Occasional use of extremities, persistent lameness.	Abnormal appearance

### 2.7 Radiographic Assessment

Radiological examinations (FDR Smart X digital Radiography System, Fujifilm, Japan) were performed once a week from the post‐operative first day until the fracture healing was completed. Radiological examination evaluated the maintenance of the anatomical position and healing levels.

## 3 Results

### 3.1 Patients Included

This study included eight cats, all crossbreeds. The cats were aged between two and five years and weighed between 2.4 and 5.9 kg. It was determined that three cases were caused by traffic accidents, three cases were caused by falling from height and two cases were caused by hitting and impacting. Closed, supracondylar distal femur fractures were identified in all cases (Table 2).

**TABLE 2:** Clinical outcomes and surgical details of distal supracondylar femoral fractures in cats treated with the EFECE system.

Case	Signalment (age, sex, bodyweight)	Aetiology	Tissue condition^a^	Fracture location	Complications	First time to use the limb	Completion of consolidation (day)	Outcome veterinary assessment^b^	Surgery time
1	2‐year‐old male crossbred, weighing 2.4 kg.	Traffic accident	Closed	R‐Supracondylar distal femoral oblique fracture	Open wound associated with the implant on the lateral side of the distal EFECE region.	1	49	Good	95 min.
2	4‐year‐old male tabby, weighing 5.9 kg.	Fall from high	Closed	R‐Supracondylar distal femoral transvers fracture	No complications.	2	35	Excellent	90 min.
3	3‐year‐old male crossbred, weighing 4.6 kg.	Hitting and impacting	Closed	R‐Supracondylar distal femoral transvers fracture	No complications.	1	42	Excellent	85 min.
4	5‐year‐old male crossbred, weighing 5.9 kg.	Traffic accident	Closed	L‐Supracondylar distal femoral transvers fracture	Oedema	1	42	Excellent	70 min.
5	2‐year‐old male crossbred, weighing 4.3 kg.	Fall from high	Closed	L‐Supracondylar distal femoral transvers fracture	No complications.	1	42	Excellent	65 min.
6	2‐year‐old male crossbred, weighing 4.3 kg.	Hitting and impacting	Closed	R‐Supracondylar distal femoral oblique fracture	No complications.	2	35	Excellent	55 min.
7	2‐year‐old female tabby, weighing 2.5 kg.	Fall from high	Closed	L‐Supracondylar distal femoral oblique fracture	Open wounds associated with the implant on the medial and lateral sides of the distal EFECE region.	2	56	Good	45 min.
8	3‐year‐old female van cat crossbreed, weighing 3.2 kg.	Traffic accident	Closed	L‐Supracondylar distal femoral transvers fracture	No complications.	1	42	Excellent	45 min.

Abbreviations: L = left, R = right.

^a^ Tissue condition: Fractures were classified as closed (intact soft tissues with no communication to the external environment) or open (fracture site exposed to the external environment with associated soft tissue damage) based on clinical and radiographic assessment.

^b^ Outcome: Final assessment of fracture healing and limb functionality, categorized as excellent, good, fair and poor.

### 3.2 Radiological Examination

**FIGURE 6:**
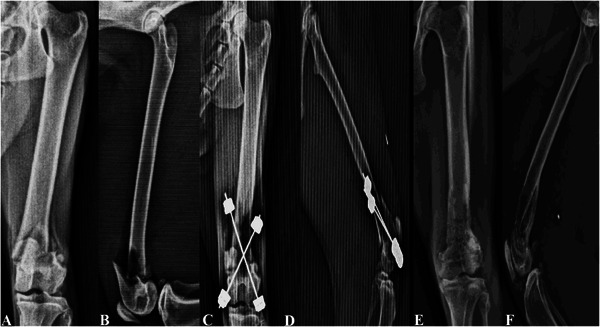
Case no. 8: X‐Ray images: (A) pre‐operative A/P, (B) pre‐operative M/L, (C) post‐operative Day 1 A/P, (D) post‐operative Day 1 M/L, (E) after the removal of EFECE system A/P and (F) after the removal of EFECE system M/L.

All eight patients were successfully treated (defined as complete fracture consolidation and full functional recovery). Radiological examinations showed that the first fibrous callus formed on the fracture line in an average of 7.8 days (between the 7th and 14th day), while fracture consolidation was completed in an average of 42.8 days (between the 35th and 56th day) (Figure 6) (Table 2). The EFECE system was removed under sedation, utilizing its percutaneous removal feature, immediately after radiographic confirmation of fracture healing. During the treatment process, no complications such as breakage of the EFECE wires or loosening of the wire holders were noticed.

### 3.3 Clinic Follow‐Up Evaluation

Clinical examinations showed that all patients began using the affected extremities within 1–2 days. In the evaluation of functional and cosmetic outcomes, six cases (Cases no. 2, 3, 4, 5, 6 and 7) were graded as good, while two cases (Cases no. 1 and 8) were graded as moderate (Table 1). As of the post‐operative seventh day, lameness symptoms disappeared in all patients except for two patients (Case no. 1 and 7), and the patients were able to perform intermittent weightbearing with their extremities (Table 2).

In one case (Case no: 4), oedema was detected on the post‐operative first day, and a cold compress was applied twice daily for 15 min, for 3 days. The oedema regressed on the fourth day. One case (Case no.: 1) had an infected wound in the lateral condyle and one case (Case no.: 7) had an infected wound in both the lateral and medial condyle due to the EFECE wire holder, which were noticed on the third post‐operative day. In these cases, diagnosed as infected wounds, swab samples were collected from the wound site and sent to the laboratory for microbiological analysis. *Escherichia coli* (Case no.: 1) and *Streptococcus canis* (Case no.: 7) were isolated from the swab samples by microbiological analysis. Based on the results of microbiological analysis, treatment in Case 1 was continued with enrofloxacin (5 mg/kg, once daily), as *Escherichia coli* was found to be susceptible to this antibiotic. In Case 7, treatment was continued with clindamycin (10 mg/kg, twice daily), as *Streptococcus canis* was susceptible to clindamycin. The wounds in these two cases (Cases no. 1 and 7) were locally dressed with rivanol solution three times a day for the first 4 days. Subsequently, dressings were continued with a mixture of hydrogel and alginate (Nu‐Gel, Systagenix, Gargrave, North Yorkshire) and rifamycin sodium (Rif, Koçak Farma, Üsküdar, İstanbul, Türkiye). The wounds healed completely on the eighth day in Case 1 and the 11th day in Case 7, respectively.

After the treatment, lameness and extremity appearance of the cases were graded as good in two cases and excellent in six cases according to the functional and aesthetic diagnosis stated by Rovesti (Table 2) (Rovesti et al. 2007). All cases were followed up for 3 months via telephone interviews conducted with the owners of the patients. During this follow‐up period, the medical conditions of all patients were found to be positive, and no lameness symptoms were observed in any of them.

## 4 Discussion

The use of orthopaedic implants in the operative treatment of supracondylar fractures has various disadvantages due to the proximity of the fracture to the joint region, the short distal fragment and the spongiform nature of the bone tissue in the fracture region (Guiot and Déjardin 2017; Harasen 2002). It has been reported that intramedullary pins, figure‐of‐eight stainless steel wires, arrow pins, crossed K‐wires and screws can be used for the stabilisation of supracondylar fractures, especially in the femur, while a more rigid fixation can be achieved with a mini plate, external fixator or stable interlocking nail (Altunatmaz et al. 2017; Rubinos and Meeson 2022; Spångberg et al. 2019; Marturello et al. 2021; Yurdakul and Sağlam 2009). Since these current methods are associated with high complication rates (Beever et al. 2017; DeCamp et al. 2016; Field et al. 2018; Karadeniz and Keskinoz 2020; Macias et al. 2006), this study aimed to evaluate the use of a new method in the operative treatment of supracondylar fractures. Accordingly, clinical and radiological findings of the EFECE system, which has been reported to provide compact fixation, fewer complications and earlier healing (Karadeniz and Keskinoz 2020), were evaluated in the treatment of distal femur fractures in cats. Thus, it was aimed to reduce the fracture healing time and the risk of major complications, as well as to contribute to the literature on the subject with a new system that can be more easily applied and removed in the treatment of related fractures.

Karadeniz and Keskinoz (2020) used the system in the treatment of patella fractures to evaluate the biomechanical behaviour of EFECE systems. In the study, simulation of a transverse fracture of the patella in human cadavers was formed, the traditional tension band method was applied in one group and the fixation method with the EFECE system was applied in the other group. The maximum force generated by applying a progressive distraction force to both groups and the total amount of elongation during this force was measured. The average maximum force was determined as 740 N (720–810 N) with EFECE systems and 330 N (240–510 N) with the tension band method. The average elongation was determined as 2.5 mm (1.6–2.5 mm) in EFECE systems and 3.4 mm (2.2–3.8 mm) in the tension band method. Consequently, it was emphasised that the EFECE system provided much more retention than traditional methods in the fixation of patella fractures and may have many areas of application in surgery due to its working principles. Based on this scientific data, the EFECE system was applied in the treatment of supracondylar distal femur fractures in eight cats. It was thought that the ability of the system to compress the fracture site would contribute to the treatment of this type of fracture.

It is known that the diameter of the Kirschner wire used as trans cortex in the operative treatment of fractures should not exceed 20% of the bone diameter (Anderson and St. Jean 1996). The study conducted by Ferretti indicated that 1.0–1.6 mm diameter wires used in cats and dogs were suitable (Ferretti 1991). In their study, Gülaydın and Alkan (2024) reported that they used 1–1.5 mm Kirschner wire and did not notice any complications in wire breakage or bone tissue. The system‐compatible Kirschner wire used in the study was 1.2 mm, and it was concluded that the diameter of the pin was suitable for the bone tissue and the weight of the patient since no complications related to the pin occurred during the treatment.

Surgical site infections (SSIs) are defined as infected wounds that develop in the post‐operative region (Weese 2008). The reported incidence of SSIs generally ranges between 3% and 10% (Eugster et al. 2004; Fitzpatrick and Solano 2010; Frey et al. 2010; Turk et al. 2015). In orthopaedic surgeries involving implants, the rate of SSIs is reported to be five to six times higher, primarily due to the role of biofilm‐forming bacterial agents in the development of antimicrobial resistance (Khatoon et al. 2018). Prophylactic antimicrobial therapy aims to prevent SSIs and reduce bacterial colonization in the tissues (Boothe and Boothe 2015). For this purpose, the use of cephalosporin‐class antibiotics combined with marbofloxacin is recommended (Valkki et al. 2020). Based on the literature, in our study all cases received post‐operative prophylactic treatment with ceftriaxone at a dose of 20 mg/kg for 10 days (Unacefin 0.5 g, Yavuz İlaç, Istanbul, Türkiye).

Early mobilization of the affected limb following fracture treatment is emphasized as crucial for preventing joint disorders and potential muscle atrophy (Patel et al. 2012; Gülaydın and Alkan 2024). In cats, the use of the affected limb after treatment of humeral and femoral fractures with various techniques has been reported to range between 1 to 3 days (Silva et al. 2012; Sancak et al. 2014; Gülaydın and Alkan 2024; Spångberg et al. 2019). Additionally, Altunatmaz et al. (2017) and Rathnadiwakara et al. (2020) reported that cats regained partial weight‐bearing ability on the affected limbs between the third and fifth post‐operative days. In this study, which evaluates a novel fixation system, all cases demonstrated functional use of the affected limb within 1 to 2 days, consistent with findings in the existing literature.

In this study, the cats completed their full functional recovery in an average of 42.8 days (between the 35th and 56th day). Altunatmaz et al. (2017), applied intramedullary two‐way crossed Kirschner wire in supracondylar and diaphyseal femur fractures in cats and reported that they were able to heal completely (sufficient callus) between 32–44 days. Gülaydın and Alkan (2024) also used hybrid external fixators in the treatment of similar fractures in cats and showed that consolidation was completed in 4 weeks in two cases, 5 weeks in two cases and 6 weeks in six cases. Another study using a modified intramedullary fixation technique in the treatment of distal femoral Salter‐Harris Type I and II fractures in cats reported that the fracture was completely healed in all cases on radiographic examination 6 to 8 weeks after surgery (Bondonny et al. 2024). These results indicated that the system used provided healing in cats at similar times to similar studies. The fact that the healing times were not very different from the study conducted with hybrid external fixators (Gülaydın and Alkan 2024), which are known as a rigid system, provides a biomechanical advantage and a tight fixation in the EFECE system due to the ability to stretch the Kirschner wires.

Various complications have been reported following the treatment of distal femur fractures with different implants (Bondonny et al. 2024). Significant disadvantages highlighted include the inability to achieve rigid fixation of fragments and pin migration in intramedullary pin applications (Bondonny et al. 2024; Karadeniz 2021), implant failure and difficulty with skin closure in plate systems (DeCamp et al. 2016; Field et al. 2018; Yurdakul and Sağlam 2009) and pin tract infections in external fixation systems (Beever et al. 2017; Gülaydın and Alkan 2024; Gülaydın et al. 2024). Spångberg et al. (2019) reported an early fixation failure in one case due to insufficient wire tensioning, necessitating revision surgery. Furthermore, in one case, lameness recurred after 4 weeks due to the breakage of a stainless steel wire. Bondonny et al. (2024) also reported minor complications (oedema) in five cases and major complications (osteomyelitis, patellar luxation) in three cases.

In their study comparing three methods, Gall et al. (2022) reported general complication rates as 10% (1 of 10 cases/1 major) in the bone plating group, 50% (8 of 16 cases/1 catastrophic, 3 major, 5 minor) in the plate‐rod construct group, and 65% (20 of 31 cases/11 major, 9 minor) in the external skeletal fixator group. In a study comparing two methods, Longley et al. reported minor complications in 14 of 22 cases (63.6%), major complications in 2 cases (9.2%) and catastrophic complications in 2 cases (9.2%) in the external fixator group. In the plate‐screw group, they encountered minor complications in 1 of 15 cases, major in 2 cases, and catastrophic in 1 case (Longley et al. 2018). When comparing the complication rates from these two studies, it is evident that more favourable outcomes were achieved in groups treated with plate‐screw fixation.

In the current study, no major complications that would hinder fracture treatment, such as breakage of EFECE wires or loosening of wire holders, were observed during the treatment period. However, minor complications (oedema, infected wound related to an EFECE wire holder) requiring no revision surgery occurred in three of eight cases (37%). In the case where oedema was observed (case no: 4), the oedema subsided with cold compress application for 3 days. In cases diagnosed with infected wounds (Case no: 1 and 7), swab samples were taken from the wound area, microbiological analysis was performed and necessary antibacterial and local wound care were applied according to antibiogram results. The wound healed completely on Day 8 in Case no.: 1 and on day 11 in Case no.: 7. The infected wound was thought to have developed due to the relatively large size of the system's wire holders available for small cats. This complication was reported to the company officials, and subsequently, information was received that new models with wire holders reduced by approximately one‐third in size had been produced. Although a relatively high overall complication rate was determined in this study due to the limited number of cases, it is believed that more accurate rates can be achieved with larger sample size studies. Furthermore, the complete absence of catastrophic or major complications in this study can be considered a positive feature of the fixation system used.

## 5 Conclusion

In the present study, supracondylar distal femur fractures of the cats in the scale included in the study were successfully treated using the EFECE system. It was observed that the extremity usage times and healing times of the cases were parallel to the rigid systems (such as the mini plate, external fixator and stable interlocking nail) in similar studies (Silva et al. 2012; Sancak et al. 2014; Gülaydın and Alkan 2024; Spångberg et al. 2019; Altunatmaz et al. 2017; Rathnadiwakara et al. 2020; Longley et al. 2018; Gall et al. 2022). It was thought that this result could be achieved due to the capability of the EFECE system to provide fixation after reduction with thin wires by compressing the fracture line and to keep the EFECE retainers in place during the treatment. Furthermore, the fact that post‐operative care was performed by the operator during the study contributed to the success of the treatment with minimal complications. It appeared that the limited incision and open reduction of the EFECE systems allowed the operator to shorten the recovery time. It was also determined that the percutaneous application of the system met the criteria required for ideal biological osteosynthesis. The limiting factors of the study were the small sample size and the fact that, in two cases with complications, the EFECE holders positioned at the distal condyle were too large relative to the patient's condylar volume (these two cases had smaller body sizes compared to the others). It was concluded that further studies involving a larger number of cases are needed and that EFECE holders should be manufactured in smaller sizes for smaller cats. Considering the results of the presented study and the features of the EFECE system, it was determined that it is important to conduct comprehensive studies by applying the system in the treatment of fractures in cats in appropriate anatomical regions. Furthermore, it was thought that it would be beneficial to expand the areas of application of the system by developing it according to the size alternatives of the cases to be used, and it would introduce novelty to the existing surgical solutions on the subject.

## Author Contributions

**Ali Gülaydin**: conceptualization (lead); methodology (lead); validation (lead); writing – original draft (lead); project administration (lead). **Nihat Şindak**: validation (equal); visualization (lead); writing – review and editing (equal). **Mustafa Barış Akgül**: supervision (lead); validation (equal); writing – review and editing (equal). **Onur Yildirim**: investigation (lead); data curation (lead); writing – review and editing (equal). **Emre karadeniz**: conceptualization (supporting); methodology (supporting); resources (lead); writing – review and editing (equal). **Müzzemil Hattap Soysal**: formal analysis (lead); writing – review and editing (equal). All authors have read and agreed to the published version of the manuscript.

## Funding

This research was funded by the Siirt University Scientific Research Projects Coordinatorship, grant number 2022‐SIÜVET‐006.

## Ethics Statement

This study was approved by the Local Ethics Committee for Animal Experiments at Siirt University with decision number 2021/04/28 on 26/11/2021. The study was conducted in accordance with the US National Research Council's guidelines for the Care and Use of Laboratory Animals.

## Conflicts of Interest

The authors declare no conflicts of interest.

## Data Availability

The data supporting the findings of this study are available within the article. Further inquiries can be directed to the corresponding author.
